# Real-World Implications of a Rapidly Responsive COVID-19 Spread Model with Time-Dependent Parameters via Deep Learning: Model Development and Validation

**DOI:** 10.2196/19907

**Published:** 2020-09-09

**Authors:** Se Young Jung, Hyeontae Jo, Hwijae Son, Hyung Ju Hwang

**Affiliations:** 1 Office of eHealth Research and Business, Seoul National University Bundang Hospital Seongnam-si Republic of Korea; 2 Department of Family Medicine, Seoul National University Bundang Hospital Seongnam-si Republic of Korea; 3 Basic Science Research Institute, Pohang University of Science and Technology Pohang Republic of Korea; 4 Department of Mathematics, Pohang University of Science and Technology Pohang Republic of Korea

**Keywords:** epidemic models, SIR models, time-dependent parameters, neural networks, deep learning, COVID-19, modeling, spread, outbreak

## Abstract

**Background:**

The COVID-19 pandemic has caused major disruptions worldwide since March 2020. The experience of the 1918 influenza pandemic demonstrated that decreases in the infection rates of COVID-19 do not guarantee continuity of the trend.

**Objective:**

The aim of this study was to develop a precise spread model of COVID-19 with time-dependent parameters via deep learning to respond promptly to the dynamic situation of the outbreak and proactively minimize damage.

**Methods:**

In this study, we investigated a mathematical model with time-dependent parameters via deep learning based on forward-inverse problems. We used data from the Korea Centers for Disease Control and Prevention (KCDC) and the Center for Systems Science and Engineering (CSSE) at Johns Hopkins University for Korea and the other countries, respectively. Because the data consist of confirmed, recovered, and deceased cases, we selected the susceptible-infected-recovered (SIR) model and found approximated solutions as well as model parameters. Specifically, we applied fully connected neural networks to the solutions and parameters and designed suitable loss functions.

**Results:**

We developed an entirely new SIR model with time-dependent parameters via deep learning methods. Furthermore, we validated the model with the conventional Runge-Kutta fourth order model to confirm its convergent nature. In addition, we evaluated our model based on the real-world situation reported from the KCDC, the Korean government, and news media. We also crossvalidated our model using data from the CSSE for Italy, Sweden, and the United States.

**Conclusions:**

The methodology and new model of this study could be employed for short-term prediction of COVID-19, which could help the government prepare for a new outbreak. In addition, from the perspective of measuring medical resources, our model has powerful strength because it assumes all the parameters as time-dependent, which reflects the exact status of viral spread.

## Introduction

Similar to the 1918 influenza pandemic that occurred more than 100 years ago, the COVID-19 pandemic has created major disruptions worldwide. At the end of World War I, the 1918 influenza pandemic wreaked havoc globally; it killed more than 40 million people, more than 2% of the world’s population [1]. During the outbreak, preventive measures such as social distancing and wearing masks were recommended to curb the spread of the virus [2]. Unfortunately, these measures were insufficient. The 1918 influenza pandemic exhibited an unusual bimodal or trimodal peak in the United States, lasting for almost two years [3]. Thus, by analogy, we can infer that decreases in infection rates of COVID-19 do not guarantee continuity of the trend. Therefore, it is necessary to develop a precise spread model of COVID-19 that responds promptly to the dynamic situation of the outbreak. If the model accurately measures the effectiveness of COVID-19–related preventative measures and provides reasonable information about the spreading trend in the next few days, it will be possible to proactively minimize damage by taking effective actions against recurring outbreak situations. Furthermore, we assessed the potential roles of a number of public health measures acting in advance based on the developed model to reduce contact rates and thereby reduce transmission of the virus in the absence of a COVID-19 vaccine [4].

Recently, there have been numerous studies on developing models to find a mathematical description of a system and translate it to the current situation of COVID-19. These studies typically introduce the susceptible-infected-recovered (SIR) model or its derivatives. In some of these studies, the model parameters are considered as constants due to the complexity of modeling. For instance, a previous study proposed a conceptual model that includes individual behavioral reactions and government actions, while another study reviewed the basic reproduction number of COVID-19 with constant parameters [5,6]. However, the reproduction number (R) innately assumes time-dependent variables. R is a function of three primary parameters; two of these are biological constants (the infectiousness of the pathogen and the duration of contagiousness after a person becomes infected), and the other is a sociobehavioral and environmental variable (the contact rate) [7]. The contact rate causes the reproduction number to fluctuate through human-to-vector or human-to-human interactions varying over time or space. Thus, it is more reasonable to define mathematical parameters in a model as time-dependent variables. However, previous representative studies did not use this method. A previous study divided the phase manually and considered the parameters as time-varying piecewise constants [6]. Other studies considered the parameters as partial functions of time and proposed methods to approximate the time-varying parameters [8,9]. More recently, a method to quantify the effects of quarantine control using a neural network was proposed. Although the authors considered the strength of quarantine control as a time-dependent parameter, the other parameters were still considered as constants [10]. Overall, most previous studies partially adopted time-variant parameters due to technical difficulties. In general, parameters of the deterministic SIR model with constant parameters can be estimated after solving the solutions of the model. However, this approach has a limitation when the model has time-dependent parameters. In previous studies related to COVID-19 [11,12], it was already recognized that parameters will change at a specific moment, such as the early phase of the epidemic, enforcement of the quarantine policy, or supply of medical equipment. As a result, piecewise constant parameters emerged depending on the artificially divided time intervals. In contrast, we suggested a new method to calculate the time-varying parameters without any artificial setting. This method enables us to analyze the times when unusual events occur and to evaluate the quarantine policy. This is the starting point of this research. We aimed to develop a model that was more precise and sensitive than previous models by introducing as many time-dependent parameters as possible to reflect that the current situation is changing on a daily basis. We adopted the SIR model with the concept of the forward-inverse problem. Furthermore, we approximated outcome variables and parameters in the model with neural networks to compute the infection rate, recovery rate, and reproduction numbers more accurately.

## Methods

### Methodological Overview

Mathematical modeling is a process that aims to find a mathematical description of a system and translate it into a relational expression. When a system (eg, an infectious disease) continuously changes over time, differential equations, which may include parameters, can be used to model it. The process of finding the parameters that best fit the given data from the system is called an inverse problem. In this study, we aimed to analyze COVID-19 spread in South Korea using the SIR model. We approximated each outcome variable (S, I, and R) and parameter (β and γ) in the model using deep learning. Moreover, to address the shortcomings of previous studies, we considered the parameters as functions of time, which allowed us to compute the infection rate, the recovery rate, and the time-dependent reproduction number, R_TD_. This approach is more interpretable because β(t), γ(t), and R_TD_ can be obtained as functions of time, and the overall dynamics of the actual data can also be obtained. We hypothesized that R_TD_ could be used as a surrogate marker to indicate the pressure on health care resources in a region. This is because the number of available beds for patients with COVID-19 in an area decreases when the infection rate increases or when the recovery rate stagnates or decreases.

Additionally, unlike in other models, such as the growth model, we do not assume any distribution type for the modeling. In the traditional growth model, the growth rate is considered as a piecewise constant function to compute the effective reproduction number. However, this assumption is not realistic in many cases, as the reproduction number can dramatically change. In contrast, our model is an appropriate solution for such problems due to its time-dependent nature. Furthermore, we provide numerical simulation results that guarantee the convergence of our deep learning approach. Finally, our methodology is applicable to many areas involving differential equations, and it can be easily implemented without a deep understanding of the model.

### Terminology

The reproduction number has several variants. The basic reproduction number (R_0_) is defined as the expected number of cases directly generated by one case in a population, assuming all individuals are susceptible to infection. Compared to R_0_, the effective reproduction number (R_t_) does not assume complete susceptibility of the population [7]. Strictly speaking, all reproduction numbers after the first date of introduction of new pathogens should be regarded as R_t_. In this study, we wanted to develop a time-dependent effective reproduction number that is a variant of R_t_; we designated this number as R_TD_.

### Data

**Figure 1:**
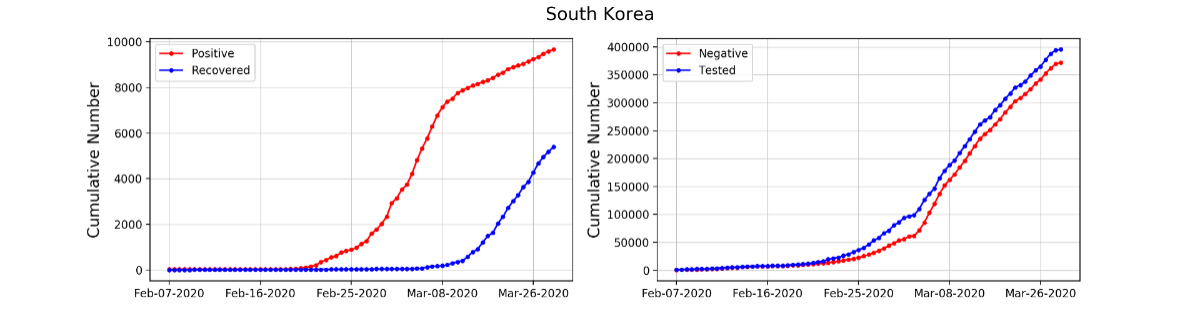
Cumulative numbers of infected and recovered COVID-19 cases in South Korea (left) and cumulative numbers of negative cases and tested people (right).

**Figure 2:**
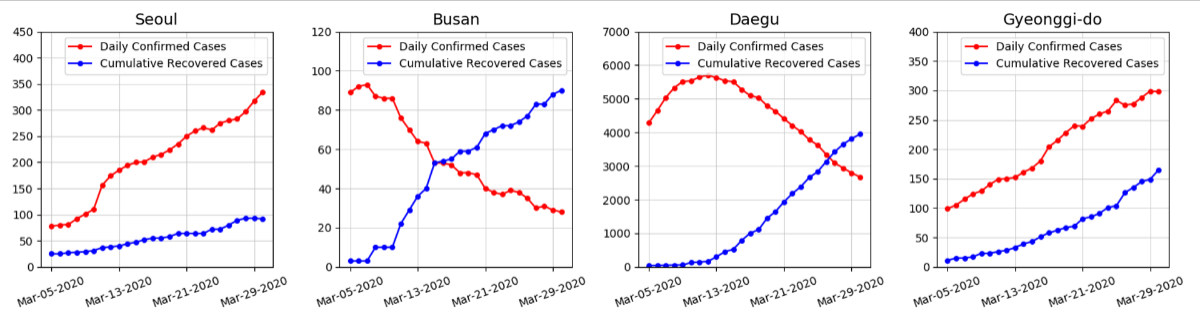
Daily numbers of confirmed cases and cumulative numbers of recovered cases for Seoul, Busan, Daegu, and Gyeonggi-do.

We collected our data from the Korea Centers for Disease Control and Prevention (KCDC) and the Center for Systems Science and Engineering (CSSE) at Johns Hopkins University. The data consisted of the cumulative numbers of tested people (*T*), confirmed cases (*I* or *I_pos_*), negative cases (*I_neg_*), and recovered or deceased cases (*R*) from February 7 to March 30, 2020, for South Korea and from March 5 to March 30, 2020, for the administrative provinces of Seoul, Busan, Daegu, and Gyeonggi. The data are available at the KCDC website [13]. Although data for South Korea are available from January 29, the numbers of negative, recovered, and deceased cases are not available for the first few weeks; therefore, we began our data range on February 7, 2020. The complete data, including numbers of negative, recovered, and deceased cases, for each administrative province are available from March 5; therefore, we used all data up to March 30, 2020. We set t=0 as March 5, 2020, when data for each province became available, and February 7, 2020 corresponds to t=–26.6 (Figures 1 and 2).

This study received an exemption from informed consent by the institutional review board committee of the Seoul National University Bundang Hospital because we used public data provided by the KCDC.

### SIR Model

**Figure 3:**
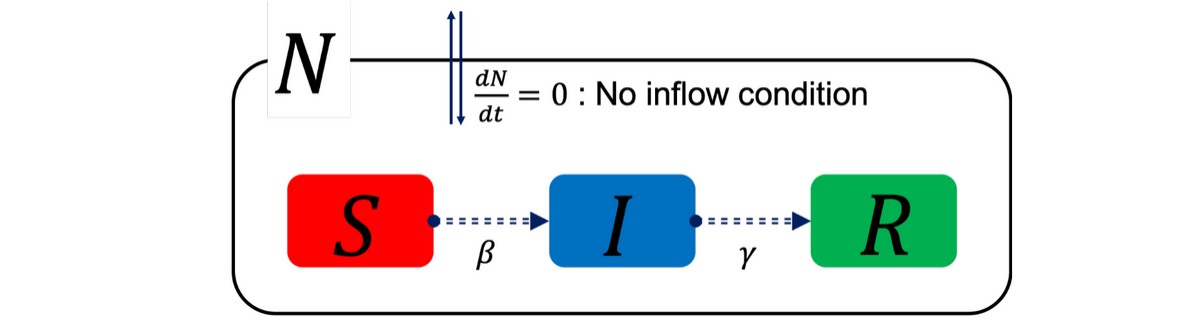
Illustration of the SIR model. I: infected; R: recovered; S: susceptible.

Infectious disease modeling in mathematics can capture an epidemic of a given infectious disease and aid public health interventions. Modeling usually requires disease-related statistical data, calculation of model parameters, and analysis of the epidemic. We adopted the SIR model, which is suitable for our data (see Figure 3). For a fixed time t≥0, let S(t), I(t), R(t), and N(t) denote the numbers of susceptible, infected, and recovered (or removed) cases and the sum of these three populations, respectively. Moreover, we applied a scaled SIR model (divided by N for each outcome variable S, I, and R) and time-varying parameters (β and γ) to the final SIR model. We also assumed that the total number of the population is time-invariant, that is, *S*(*t*) + *I*(*t*) + *R*(*t*) = 1. The mathematical formula of the SIR model is provided in detail in Multimedia Appendix 1.

### Deep Learning

**Figure 4:**
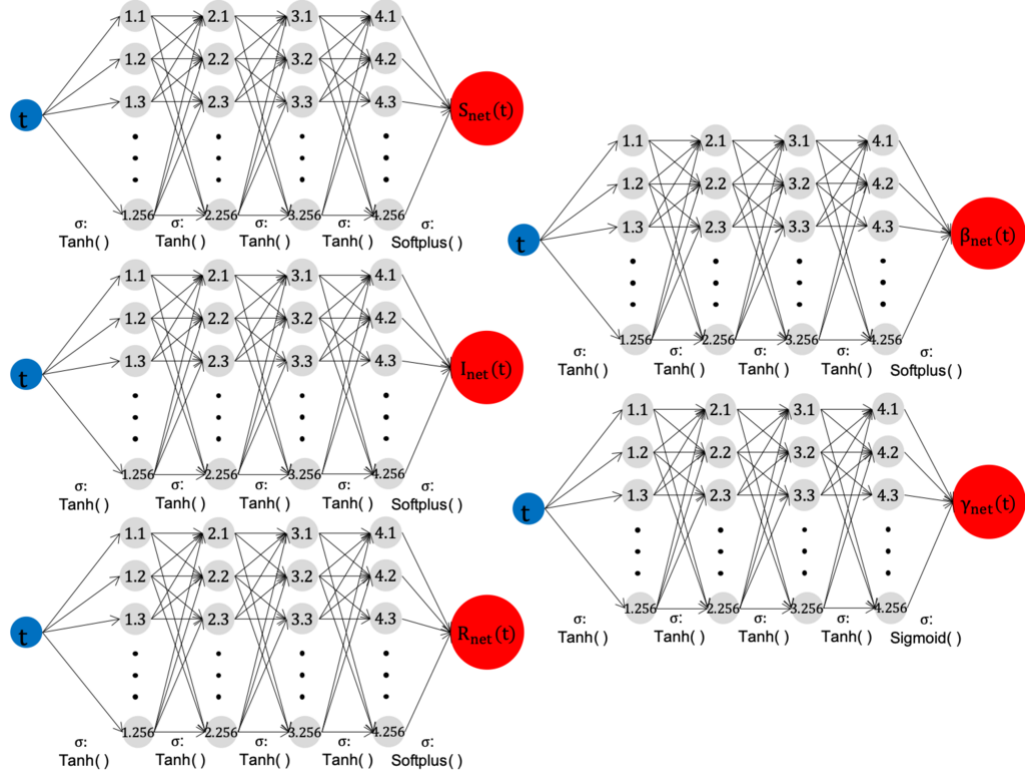
Forward-Inverse SIR model networks. Each network contains 1 input node (time t), one1output node (value), 4 hidden layers, and 256 nodes in each hidden layer. The hyperbolic tangent tanh(x) is used in the activation functions except for the last layer. The Softplus and Sigmoid functions are used in the last hidden layer to meet the constraints S, I, R, β>0, and 0<γ<1. I: infected; R: recovered; S: susceptible.

We constructed five neural network models for S, I, R, β, and γ, denoted by S_net_, I_net_, R_net_, β_net_, and γ_net_, respectively. The concrete model structures are presented in Figure 4. We applied similar training methods to solve forward and inverse problems, as introduced in previous studies [14,15]. The detailed deep learning methodology is provided in Multimedia Appendix 1.

We conducted simulations for four provinces: Seoul, Gyeonggi-do, Busan, and Daegu. We applied five deep neural network (DNN) models to derive the parameters S_net_, I_net_, R_net_, β_net_, and γ_net_. For a more accurate evaluation of the model parameters, we also provided a numerical solution called Runge-Kutta fourth order (RK4) using the estimated parameters. RK4 is one of the most well-known and theoretically proven algorithms that converges to analytic solutions. In contrast, the neural network-based methodology of this study has a weak theoretical background for convergence. Therefore, we aimed to show how close the time-dependent parameters found by DNN are to the actual solution through RK4.

For the RK4 method, we set a step size of h=10^−3^, with 26 observations used for Seoul, Busan, Daegu, and Gyeonggi and 77 observations used for South Korea. The observations are presented in Multimedia Appendix 1.

## Results

### Estimating the Parameters of the SIR model with DNN

**Figure 5:**
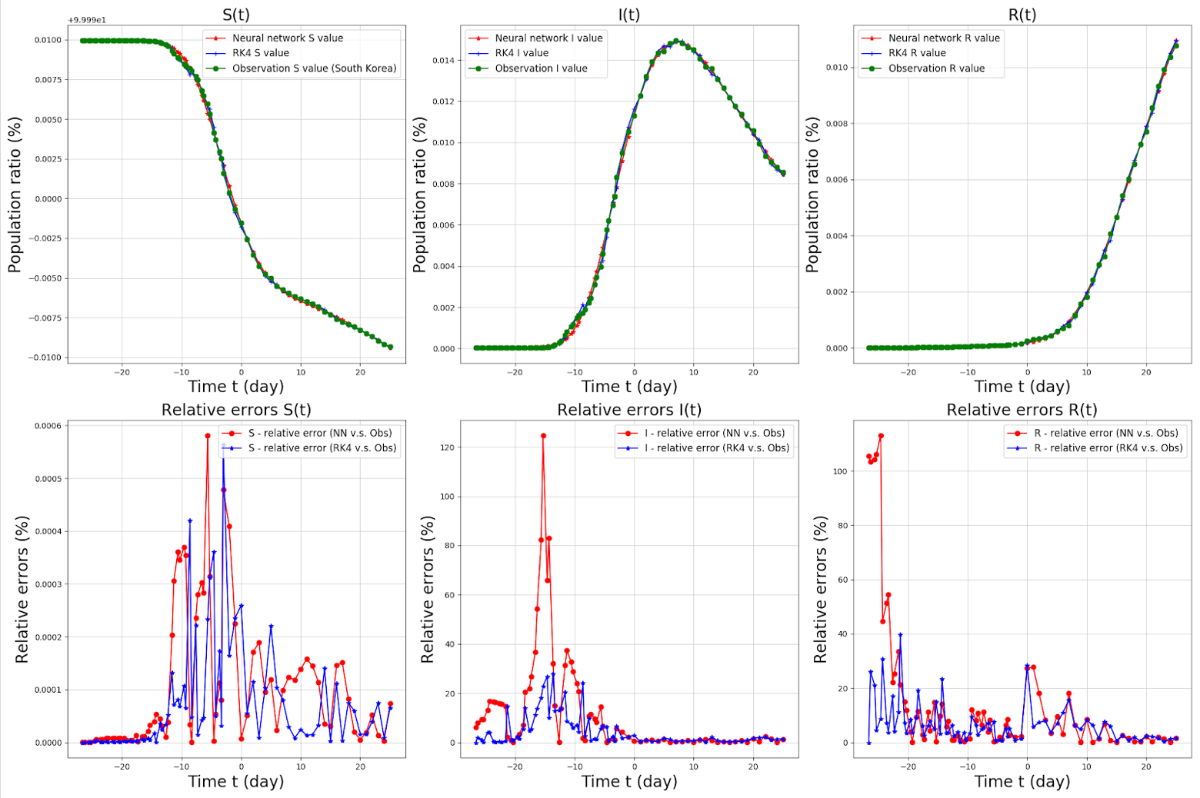
SIR model target values and relative errors from February 7 (t=–26.6) to March 30 (t=25.0), 2020, in South Korea. The red lines in the top three graphs denote the S^net^, I^net^, and R^net^ values for each graph, the green lines in the top and middle three graphs denote the observations, and the blue lines in the middle three graphs denote the RK4 results with the parameters β^net^ and γ^net^. The population ratio is the number of people in each group (S, I, and R) divided by the total number of people (N). Relative errors were defined as (|observed value − Network [or RK4] value|/|observed value|) × 100 and were calculated for each parameter. I: infected; R: recovered; RK4: Runge-Kutta fourth order method; S: susceptible.

We estimated the model parameters (β and γ) and outcome variables (S, I, and R) in the SIR model via DNNs for South Korea, Seoul, Busan, Daegu, and Gyeonggi. The results for South Korea are presented in Figure 5. The results for Seoul, Busan, Daegu, and Gyeonggi are provided in Multimedia Appendix 1 (Figures SM1 to SM4, respectively). 

**Figure 6:**
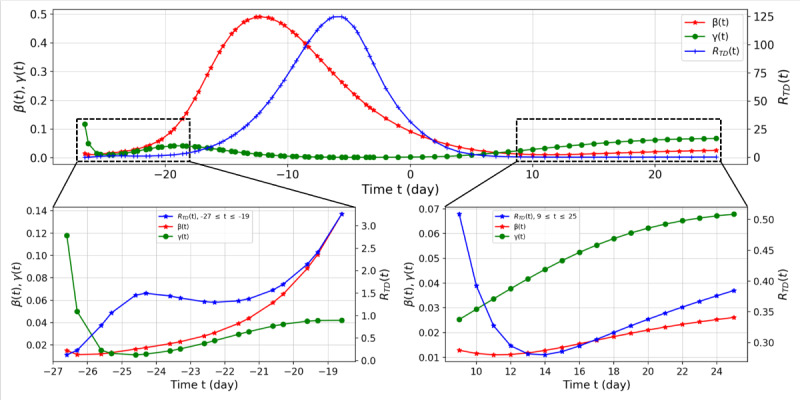
Suspected-infected-recovered model parameter network values and R_TD_ values from February 7 (t=–26.6) to March 30 (t=25.0), 2020, for South Korea. We divided the range of R_TD_ into two parts, shown at bottom left (–26.6≤t≤–19) and bottom right (9≤t≤25.0). On February 18 (t=–15.3), the first case was confirmed to be related to Shincheonji, which was the starting point of the outbreak in Daegu.

We also estimated R_TD_ for South Korea (Figure 6). 

We summarized the overall trend by analyzing R_TD_ (t). First, on February 8 (t=–25.3) in South Korea, R_TD_=1.0610 implies the spread of COVID-19. Starting from February 18, R_TD_ (t) increased dramatically (t=–15.6), and it reached its peak (R_TD_ (t)=124.8454) on February 28 (t=–5.6). After March 13 (t=8.0), R_TD_ decreased below 1 again, signaling a decreasing trend in the spread of COVID-19 from an epidemiological viewpoint. However, R_TD_ began to increase again from March 19 (t=14.0). From February 7 to March 30, the average values of β and γ were 0.1656 and 0.0253, respectively.

In the second case, Seoul, up to March 9 (t=4), β reached 0.2306 while γ only reached 0.0192, resulting in a maximum value of 12.0405 for R_TD_ in this period. After March 16 (t=11), R_TD_ decreased to 3.1244 but then increased again, reaching 3.8255 on March 30 (t=25). This indicates that effective control of the spread of COVID-19 was not achieved. The average values of β and γ were 0.0705 and 0.0140, respectively. In the third case, Busan, on March 5 (t=0), at the beginning of the observation, β was 0.1300 (Supplementary Table), while R_TD_ was 156.7965. This is because R(t), the recovery group, did not change in the initial stage, whereas γ was estimated to be 0.0008 due to the constraint γ>0. On March 8 (t=3), R_TD_ was 0.0908 because of the change in R(t), reaching 0.5401 on March 30 (t=25). The average values of β and γ were 0.0253 and 0.0670, respectively. In the final case of Daegu, similar to Busan, R_TD_ was 521.9075 at the beginning of the observation on March 5 (t=0). After March 11 (t=6), the recovery rate γ began to increase faster than the infection rate β, with R_TD_ having its lowest value of 0.1224 on March 24 (t=19). After March 24, R_TD_ increased, reaching 0.2409 on March 30 (t=25) (see Figure SM3 in Multimedia Appendix 1). The average values of β and γ were 0.0191 and 0.0387, respectively. The results for other provinces are presented in figures and tables in Multimedia Appendix 1.

### Time-Dependent Effective Reproduction Number (RTD)

**Figure 7:**
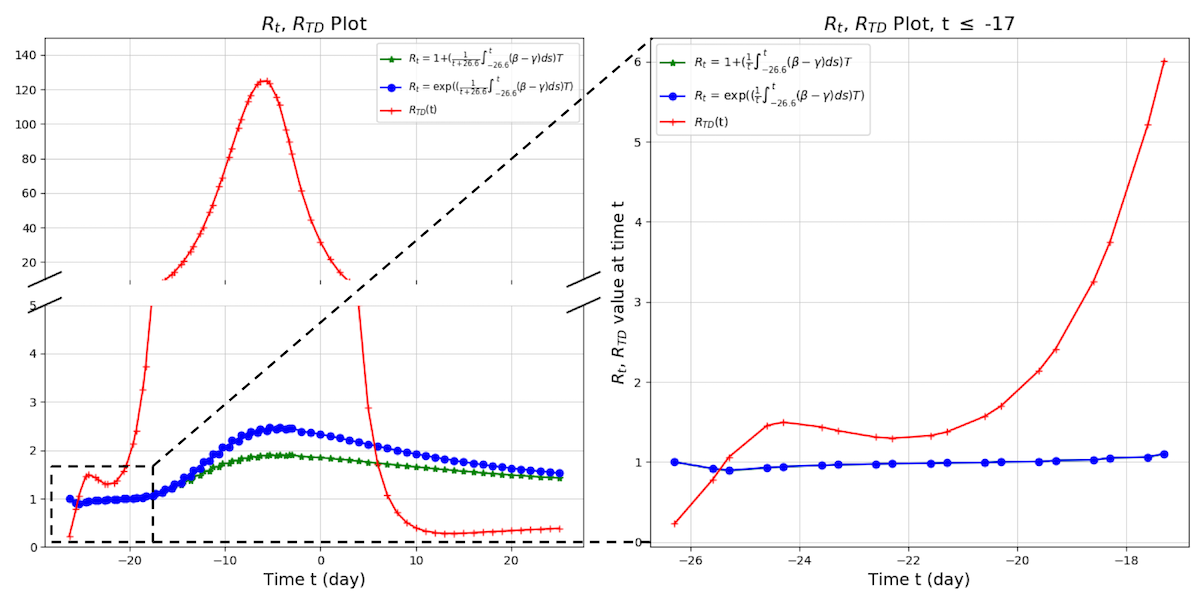
Comparison of R_TD_ and R_t_ for South Korea. R_t_ was computed based on the growth model. R_t_: effective reproduction number; R_TD_: time-dependent effective reproduction number.

Because R_TD_ is the ratio of β(t) to γ(t), R_TD_ can have a large value when γ is small compared to β. This situation can be observed in the early stage of COVID-19 spread in South Korea, excluding Seoul and Busan (eg, the Shincheonji cult cases). However, following the computation of the basic reproduction number in a previous study, we obtained the effective reproduction number R_t_ in the usual range found in previous studies [16]. In the SIR model, we approximated S as 1 because S was sufficiently large compared to I. The detailed formula is presented in Multimedia Appendix 1. R_TD_ responded more sensitively than R_t_ to the real-world situation from t=–26 to t=–18 (right side of Figure 7). 

### Characteristics of RTD

**Figure 8:**
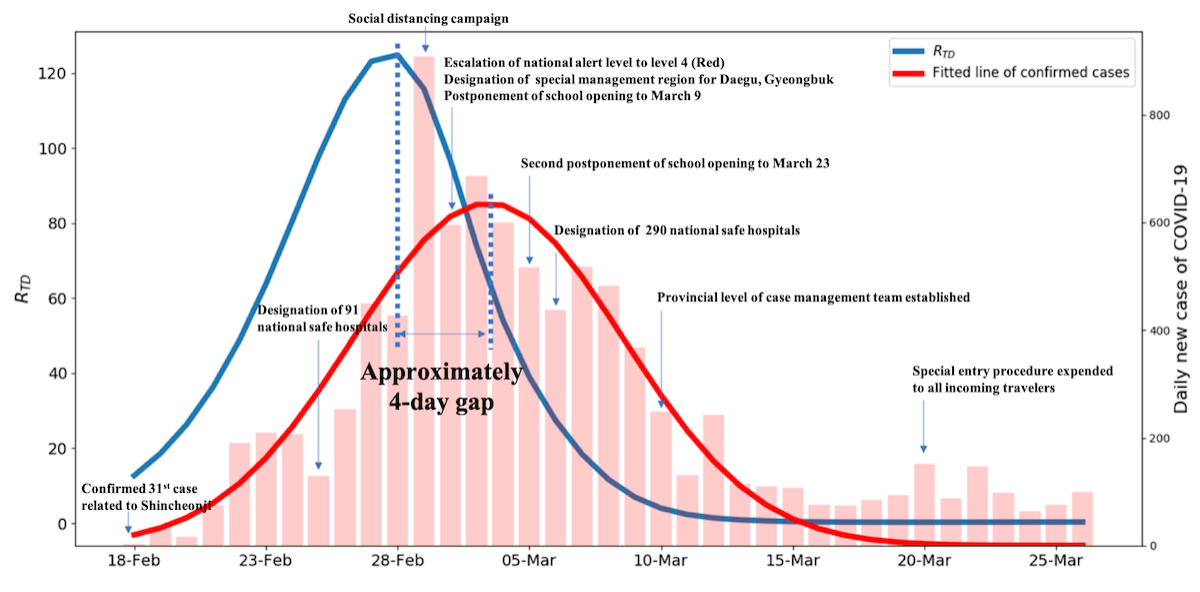
Comparison of R_TD_ and real-world confirmed cases. R_TD_: time-dependent effective reproduction number.

R_TD_ is a more sensitive and responsive marker than R_t_, and it reflects subtle changes of situations over time. Especially at the starting point of an outbreak, we can detect increasing trends more accurately with R_TD_ (Figure 7). Furthermore, R_TD_ is an indicator that precedes real-world changes. Looking at the real-world data, there is a time delay of 4 days between the peak of R_TD_ and the peak of confirmed cases (Figure 8) [17]. We also observed this pattern of time delay between the peak of R_TD_ and the peak of confirmed cases in other countries (Multimedia Appendix 1, Characteristics of R_TD_).

### Real-World Implications of RTD

The R_TD_ we developed has important real-world implications for measuring the current status of the viral spread and the effectiveness of interventions. By setting the infection rate and recovery rate as time-dependent parameters, it is possible to accurately evaluate the pressure of depletion of health resources on the community. Indeed, after March 5, when R_TD_ exceeded 500, Daegu was in danger of total depletion of medical resources [18,19]. Patients who were self-isolating at home while waiting for hospitalization died, and a previously secured negative pressure room became full and could not continuously accept severely ill patients. In response to this situation, the Korean government opened the Community Treatment Center (CTC) to care for patients with mild illness in Daegu in early March [20]. The CTC was staffed with seven physicians, five nurses, and several paramedic workers who monitored and cared for low-risk patients with COVID-19. The government would have been able to preemptively enact drastic policies if it had observed the changes in R_TD_ that preceded the trend of confirmed cases by approximately 4 days without any sacrifice of patients (Figure 8).

**Figure 9:**
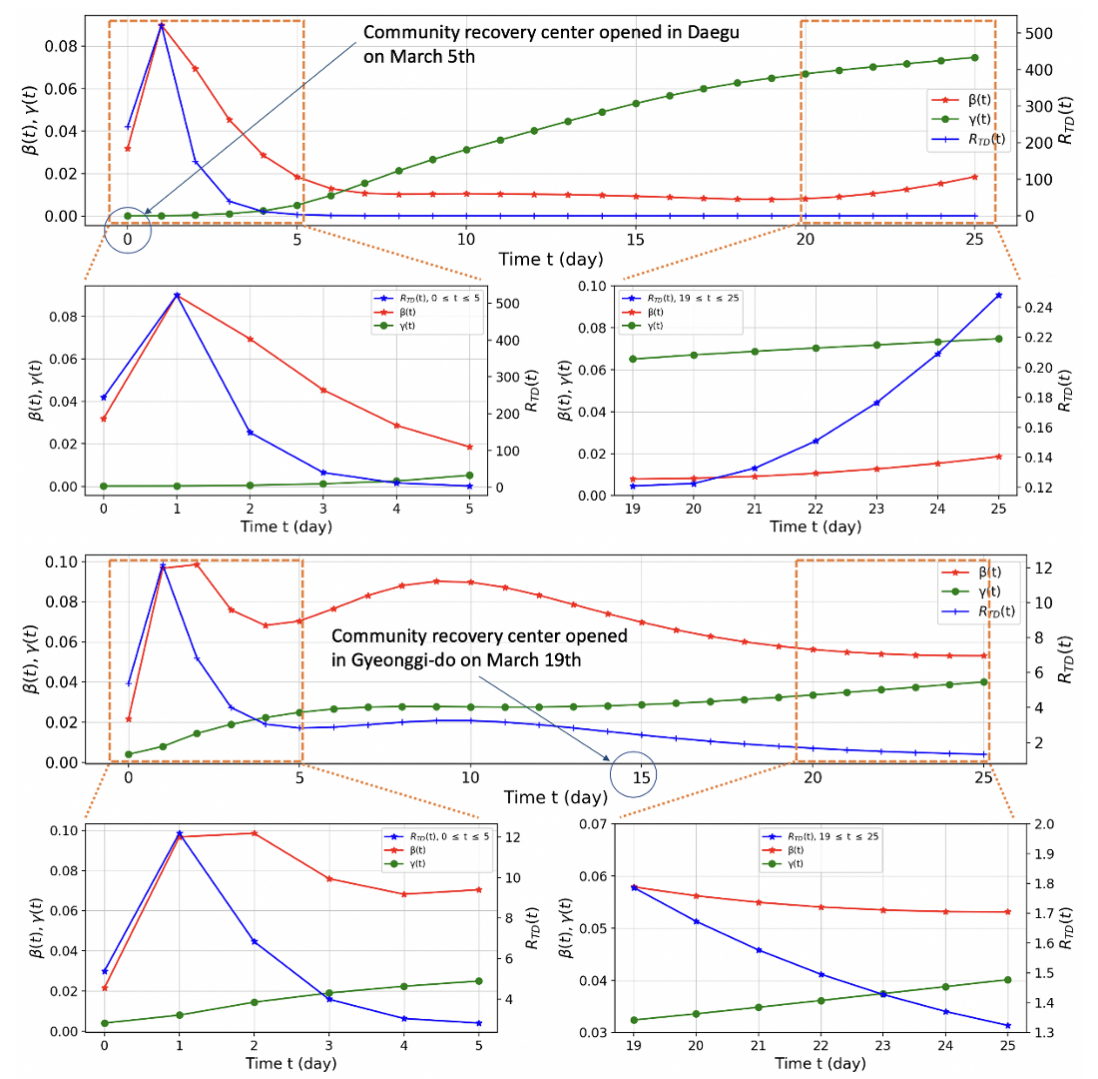
Comparison of β(t), γ(t), and R_TD_ in Daegu and Gyeonggi-do. Note the differences in the vertical scales of R_TD_. R_TD_: time-dependent effective reproduction number.

Compared to Daegu, Gyeonggi-do intervened more proactively. The R_TD_ of Daegu at the first opening of the CTC was over 500; however, that of Gyeonggi-do was 2.6. The local government in Gyeonggi-do, which closely monitored the situation in Daegu, prevented the exhaustion of medical resources by providing optimal medical services for each risk group of patients with COVID-19 in cooperation with the central government, along with general policies such as public disclosure of mobile routes of infected people, encouragement of social isolation, and wearing of masks (Figure 9).

## Discussion

### Novelty of the Model

We developed an entirely new SIR model with time-dependent parameters via deep learning methods. Furthermore, we validated the model with the conventional RK4 model to confirm its convergent nature. In addition, we evaluated our model based on real-world data reported by the KCDC, the Korean government, and news media.

Compared to previous studies, this research has the following three technical advantages. First, previous studies only dealt with the infected cases under certain assumptions, such as the cumulative number of infected cases increasing exponentially [21]. In our method, we can compute the effective and time-dependent reproduction numbers without any assumptions. Moreover, we computed the entire dynamics for S, I, and R simultaneously; therefore, the analysis is more precise. Secondly, in another previous study, the authors manually divided the phase of COVID-19 spread according to the preventative and control measures to overcome the limitation of the constant reproduction number [11]. In our method, however, we did not need to artificially divide the phases because the results including S, I, and R and the parameters are naturally time-dependent. Thirdly, rather than using statistical inference techniques as in previous research, we applied a neural network to solve the forward-inverse problem consisting of the SIR model and its parameters [9]. Therefore, our method gives deterministic and more accurate values without any statistical uncertainty. Furthermore, by leveraging the neural network, our method can capture richer structures in the data and SIR model compared to the filtering techniques used in prior research [8].

### Implications for Real-World Intervention

In the situation of a novel virus pandemic, it is crucial for every central and local government to maintain appropriate medical resources in readiness for unexpected penetration of the new disease. South Korea saw one of the most disastrous outbreaks of COVID-19 during the first few weeks of March 2020. In Daegu especially, the entire local medical system was on the brink of collapse. However, the Korean government soon developed a preemptive policy for each local government by learning from the situation in Daegu. The government solved its acute hospital bed shortage by revising the triage criteria more than seven times and implementing CTCs all over the country. Since then, lives were saved by reserving beds for the most acutely ill patients with COVID-19 and placing patients with less severe disease in CTCs [22].

 In a country such as Korea, where there is no interregional blockade, the spread of the virus can be exacerbated in a few days due to movement of the virus across regions. In fact, the number of COVID-19 cases started increasing again from March 19 (t=14.0), indicating that the containment of COVID-19 cannot be realized without achieving herd immunity or developing therapeutics.

Furthermore, we require a tool that can monitor virus outbreaks simultaneously across regions in the shortest time span.

The same principle applies even if we broaden our view from the spread of viruses between regions to the spread among countries. In the current COVID-19 pandemic, the world must work together to prevent the spread of the virus. This is because the entire world is socially, culturally, and economically intertwined through advanced transportation. Therefore, there is an urgent need for a tool that can respond sensitively over time, provide information about the current virus outbreak, and evaluate the effectiveness of interventions. The methodology and new model of this study could be employed for proactive intervention. In addition, from the perspective of measuring medical resources, our model has powerful strength because it assumes all the parameters as time-dependent, which reflects the exact status of viral spread. Furthermore, the methodology and modeling approach are scalable and universal; therefore, they can be applied to other new infectious disease pandemics if real-world data are available.

### Limitations

This research has several limitations. First, the time-dependent model of this study was validated only with COVID-19 data from South Korea. However, this model can be easily applied to data from another outbreak because the modeling process and methodology are disclosed fully in this article. To crossvalidate our strategies, we provide results of similar analyses of outbreaks in Italy, Sweden, and the United States in Multimedia Appendix 1 (see Figures SM5 to SM10). Secondly, because of the nature of deep learning, the results of the model may have been overfitted to South Korean data. However, with the new approach of this research, it is more feasible and reasonable for every researcher to adopt the modeling methodology and apply the model by training it with local data that reflect local situations. In this case, an overfitted model can be reinterpreted as a model that is appropriately fitted to the local situation or that reflects the characteristics of the region.

## Appendix

Multimedia Appendix 1 Mathematical formulation of the SIR model and supplemental figures.

## Abbreviations

CSSE: Center for Systems Science and Engineering
CTC: Community Treatment Center
DNN: deep neural network
KCDC: Korea Centers for Disease Control and Prevention
R: reproduction number
R_0_: basic reproduction number
R_t_: effective reproduction number
R_TD_: time-dependent effective reproduction number
RK4: Runge-Kutta fourth order
SIR: susceptible-infected-recovered
