# Supplementary material for: Real-World Implications of a Rapidly Responsive COVID-19 Spread Model with Time-Dependent Parameters via Deep Learning: Model Development and Validation
Source: J Med Internet Res. 2020 Sep 9;22(9):e19907. doi: 10.2196/19907 (PMC7486001; doi:10.2196/19907)
Supplement: Multimedia Appendix 1 [file jmir_v22i9e19907_app1.docx]

# Supplementary Material

## Mathematical formulation of the SIR model

We can write the scaled SIR model with time varying parameters as the following:

$\frac{dS}{dt}=-\beta(t)SI,$ (S.M.1)

$\frac{dI}{dt}=\beta(t)SI-\gamma(t)I,$ (S.M.2)

$\frac{dR}{dt}=\gamma(t)I,$ (S.M.3)

where $\beta(t)>0$ and $0<\gamma(t)<1$ denote the average number of contacts per person per time and the rate of recovery (or decease), respectively. In general, we set the initial condition $S(0), I(0),$ and $R(0)$ as the first observation data, and for the analysis, we assume that the total number of population is time-invariant, that is, $S(t)+I(t)+R(t)=1$.

##

## Deep learning: Deep neural network

Deep learning is an on-linear function approximation method using the DNN architecture. The DNN architecture consists of one input and output layer, and several hidden layers. Each adjacent layer is connected by an affine transformation followed by a non-linear activation function. Historically, the neural network model was first introduced in [(1)](https://paperpile.com/c/7DpnHz/ZOAW) and Cybenko proved that a neural network with a single hidden layer can approximate any continuous function under some conditions on the activation function. [(2)](https://paperpile.com/c/7DpnHz/ib8o) Hornik-Stinchcombe-White also showed multilayer feedforward neural networks with sigmoid activation functions can approximate any measurable function. [(3)](https://paperpile.com/c/7DpnHz/WURf) Later, Li proved that a neural network with one single layer can simultaneously approximate any measurable function and its partial derivatives on a compact set.[(4)](https://paperpile.com/c/7DpnHz/odWC)

To be more specific, let ${z_{i}}^{l}$be the value of $i^{th}$neuron in the $l^{th}$layer. Then,

${z_{j}}^{l+1}=\sum_{i\leq m_{l}} {w_{ji}}^{l+1}\sigma_{l}({z_{i}}^{l})+{b_{j}}^{l+1},$ (S.M.4)

where

$m_{l}$: the number of neurons in $l^{th}$layer

$\sigma_{l}$: the activation function in $l^{th}$layer

${w_{ji}}^{l+1}$: the weights between $i^{th}$ neuron in $l^{th}$ layer and $j^{th}$ neuron in ${(l+1)}^{th}$layer

${b_{j}}^{l+1}$: the bias of $j^{th}$ neuron in $l^{th}$ layer

## Forward-Inverse problem of differential equations

This section provides a method for solving differential equations using DNN. A system of ordinary differential equations can be represented by

$\frac{dU}{dt}=A_{p}(U),$ (S.M.5)

$U(0)=U_{0},$ (S.M.6)

where $U:[0,T]\to\mathbb{R}^{n}, n\geq1$ denotes the solution defined on a time interval $[0,T]$with a final time $T>0$, and $U_{0}$is an initial value that is given to solve the above system. The operator $A_{p}:\mathbb{R}^{n}\to\mathbb{R}^{n}$ can be modeled from a given system model and $p$ is a parameter which is contained in the operator $A_{p}$. In case of the SIR model (S.M.1)-(S.M.3), for example, the operator is given by $A_{p}(S,I,R)=(-\beta(t)SI, \beta(t)SI-\gamma(t)I, \gamma(t)I$ with parameter $p=(\beta(t), \gamma(t))$.

Once the model (S.M.5)-(S.M.6) is given, we compute the solution $U$ (forward problem) and the model parameter $p$ which best fit the data. We propose a suitable method that solves forward and inverse problems at the same time. The key idea is to utilize the DNN as the outcome variables and model parameters, and to train the networks through the model (S.M.5)-(S.M.6).

The forward problem, solving differential equations using a neural network, has been extensively studied. A neural network consists of a single layer and ten units were studied in [(5)](https://paperpile.com/c/7DpnHz/sBub) , and the results were extended in [(6)](https://paperpile.com/c/7DpnHz/nvlg) with a geometrical complex boundary. Berg-Nyström used a DNN to solve the boundary value problems in 2D linear advection and diffusion equations in [(7)](https://paperpile.com/c/7DpnHz/8Eq2). More recently, Hwang, et al applied the DNN architecture on the kinetic Fokker-Planck equation with various boundary conditions, and showed that the DNN solutions naturally admit the physical properties that the solution must satisfy. [(8)](https://paperpile.com/c/7DpnHz/nWLh)

On the other hand, the research to solve the inverse problem using a neural network has emerged more recently. In other previous study, Raissi-Perdikaris-Karniadakis tried to connect the model parameters to DNN solutions of the incompressible Navier-Stokes equation using simulation data.[(9)](https://paperpile.com/c/7DpnHz/T4OoR) Later, Jo, et al gave a mathematical reason that neural networks converge to analytic solutions in forward and inverse problems for simple partial differential equations with constant parameters. Several researches (related to COVID-19) show that having time-varying parameters is more useful . This paper follows the method that was introduced in [(10)](https://paperpile.com/c/7DpnHz/uafG). Shortly, we design both outcome variables and parameters as a function of time by DNNs for (S.M.5)-(S.M.6) and denote the DNNs by $U_{net}, p_{net},$ respectively. Therefore we construct five neural networks models for $S, I, R, \beta, \gamma$ and denote by $S_{net}, I_{net}, R_{net}, \beta_{net}, \gamma_{net},$ respectively. We also present concrete model structures in Figure 4.

## Loss functions and optimization

Note that the fully-connected DNN (S.M.4) contains a number of weights w and biases b, called network parameters. This section introduces a way to find suitable network parameters. Intuitively, one can say that $U_{net}$ is close to the solution of (S.M.5)-(S.M.6) if $U_{net}$ satisfies (S.M.5)-(S.M.6). This insight can be proven when the existence and uniqueness of the solution to (S.M.5)-(S.M.6) with given smooth parameters are given (see, for example, [(10)](https://paperpile.com/c/7DpnHz/uafG)). Since those are guaranteed for the SIR model (S.M.5)-(S.M.6), we omit the details here. Therefore, we define the following loss function (S.M.7) that can measure how similar $U_{net}$ is to the original solution. We also set up a loss function (S.M.8) to learn $p_{net}$ through the observed data at the same time. For the fixed final time $T>0$, we define

$LossDE_{T}((w_{U},w_{p}), (b_{U},b_{p}))$

$:=\frac{1}{N_{grid}}\sum_{1\leq j\leq N_{grid}}|\frac{dU_{net}}{dt}(t_{j};w_{U},b_{U})-A_{p_{net(t_{j};w_{p},b_{p})}(Unet(t_{j},w_{U},b_{U}))}|,$ (S.M.7)

$LossOBS_{N_{obs}}(w_{U},b_{U}):=\frac{1}{N_{obs}} \sum_{1\leq n\leq N_{obs}}|U_{obs}(t_{n})-U_{net}(t_{n};w,b_{U})|,$ (S.M.8)

$Loss_{T,N_{obs}}(w,b):=LossDE_{T}((w_{U},w_{p}), (b_{U},b_{p}))+LossOBS_{N_{obs}}(w_{U},b_{U}),$ (S.M.9)

where $0\leq t_{1}<t_{2}<...<t_{N_{grid}}\leq T, t_{n}, n=1,2,..., N_{obs}$ is the observation time, $U_{obs}(t_{n})$ is the observation value at time $t_{n}$, and the positive integer $N_{obs}$ is the number of total observations. We denoted the network parameters for $U_{net},p_{net}$ as $(w_{U},b_{U}), (w_{p},b_{p}),$ respectively. Notice that, in general, since the initial condition (S.M.6.) can determine from the observation data, so that it can be contained in the loss function (S.M.8). That is, it is not necessary to add a separate loss term for (S.M.6). The model was built with PyTorch (*Pytorch: An imperative style, high-performance deep learning library*) in the python environment. To find the minimizer of the loss function (S.M.9), we used the ADAM optimizer, (*Adam: A method for stochastic optimization*), one of the most popular in deep learning communities.

## Results for Seoul, Busan, Daegu, and Gyunggi-do


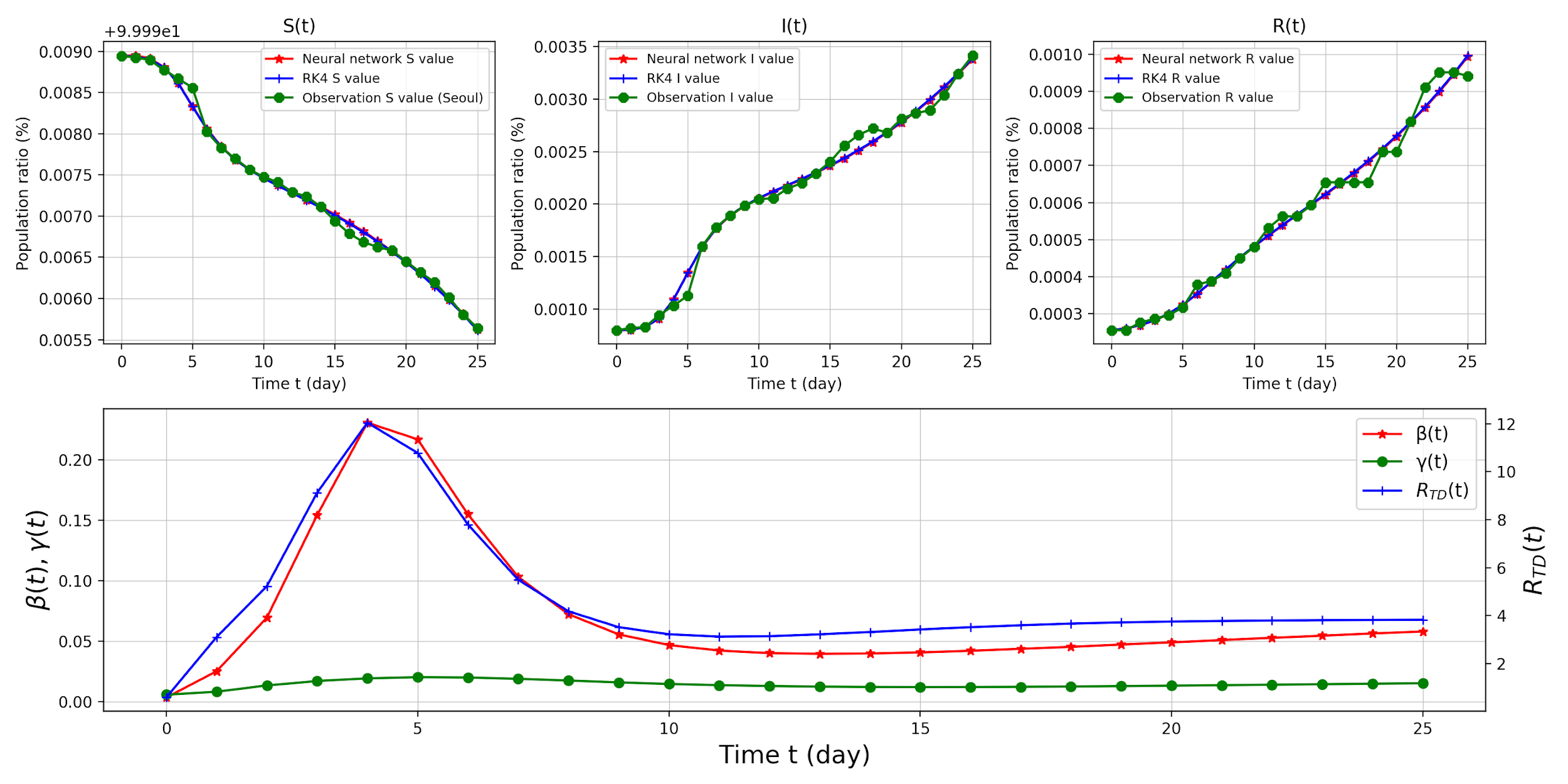


### Figure SM1. First row : SIR-model target values and relative errors. From 5, March 2020 (t = 0) to 30, March 2020 (t = 25.0) in Seoul. Red lines denote $S_{net}, I_{net}, R_{net}$ values for each graph, Green lines denote the observations, and Blue lines denote the RK4 results with the parameters $\beta_{net}$, and $\gamma_{net}$. Second row : SIR-model Parameter network values and R_TD_. From 5, March 2020 (t = 0) to 30, March 2020 (t = 25.0) in Seoul.


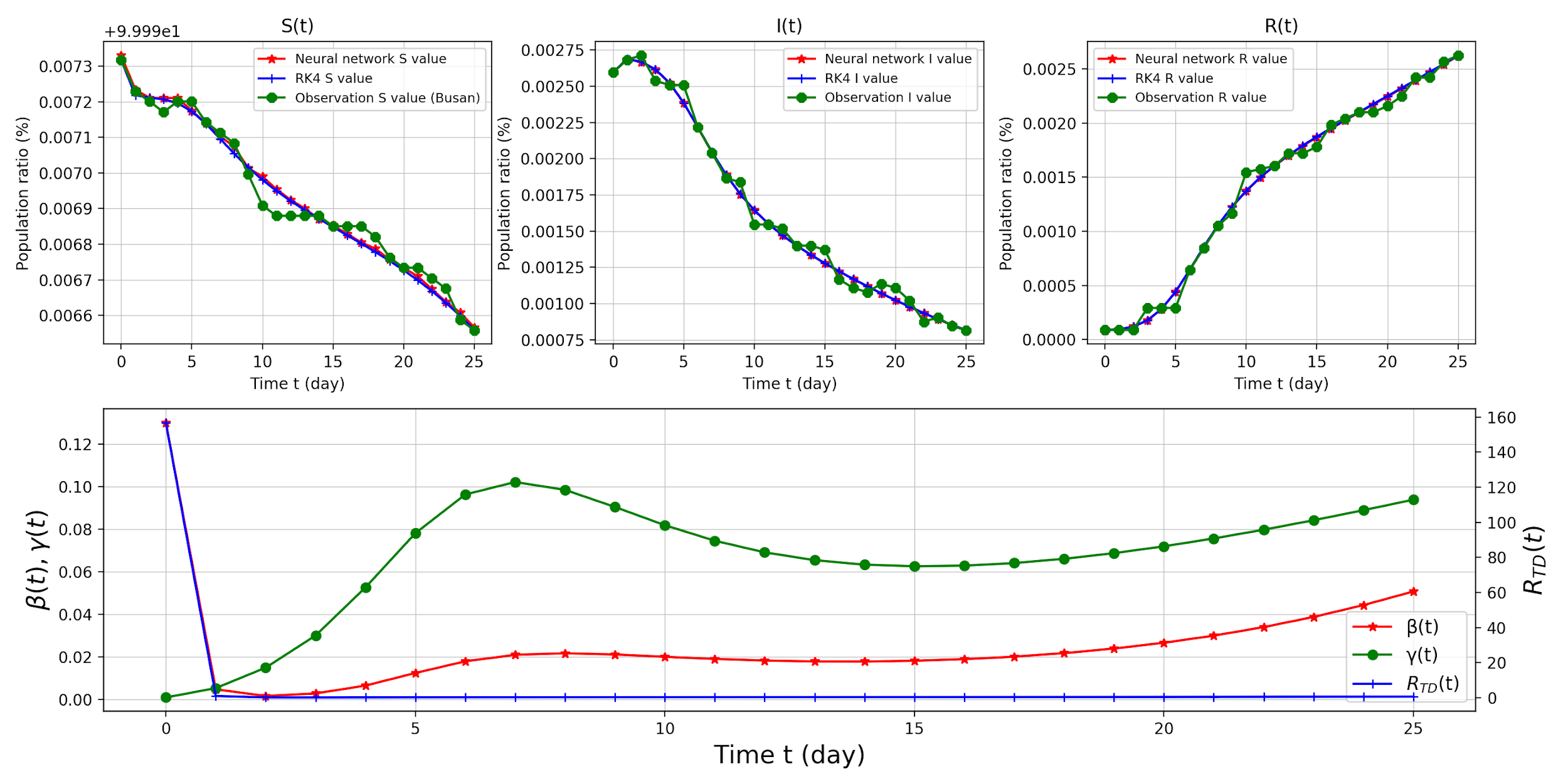


### Figure SM2. First row : SIR-model target values and relative errors. From 5, March 2020 (t = 0) to 30, March 2020 (t = 25.0) in Busan. Red lines denote $S_{net}, I_{net}, R_{net}$ values for each graph, Green lines denote the observations, and Blue lines denote the RK4 results with the parameters $\beta_{net}$, and $\gamma_{net}$. Second row : SIR-model Parameter network values and R_TD_. From 5, March 2020 (t = 0) to 30, March 2020 (t = 25.0) in Busan.


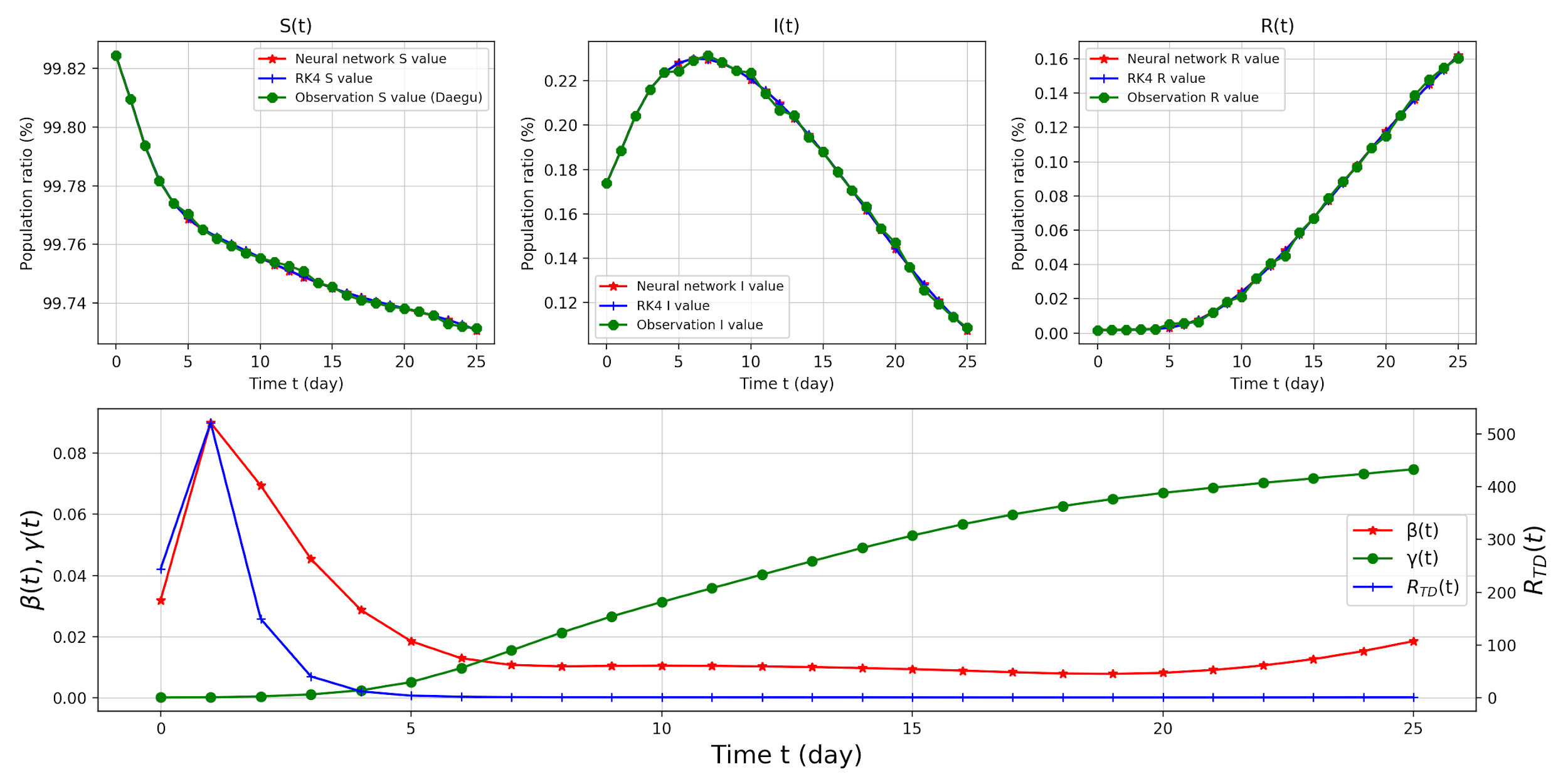


### Figure SM3. First row : SIR-model target values and relative errors. From 5, March 2020 (t = 0) to 30, March 2020 (t = 25.0) in Daegu. Red lines denote $S_{net}, I_{net}, R_{net}$ values for each graph, Green lines denote the observations, and Blue lines denote the RK4 results with the parameters $\beta_{net}$, and $\gamma_{net}$. Second row : SIR-model Parameter network values and R_TD_. From 5, March 2020 (t = 0) to 30, March 2020 (t = 25.0) in Daegu.


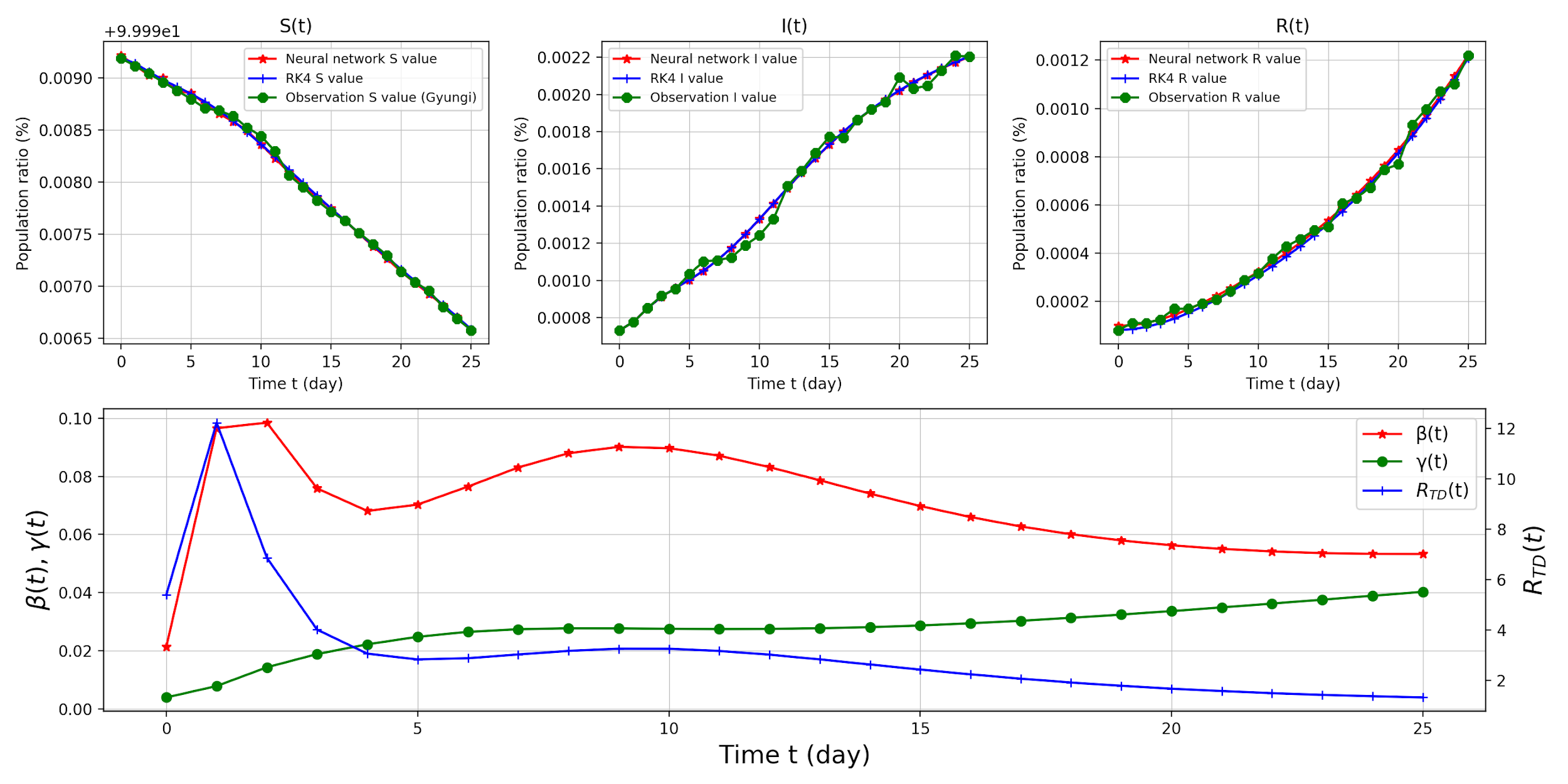


### Figure SM4. First row : SIR-model target values and relative errors. From 5, March 2020 (t = 0) to 30, March 2020 (t = 25.0) in Gyunggi-do. Red lines denote $S_{net}, I_{net}, R_{net}$ values for each graph, Green lines denote the observations, and Blue lines denote the RK4 results with the parameters $\beta_{net}$, and $\gamma_{net}$. Second row : SIR-model Parameter network values and R_TD_. From 5, March 2020 (t = 0) to 30, March 2020 (t = 25.0) in Gyunggi-do.

## Results for Italy, Sweden, and US


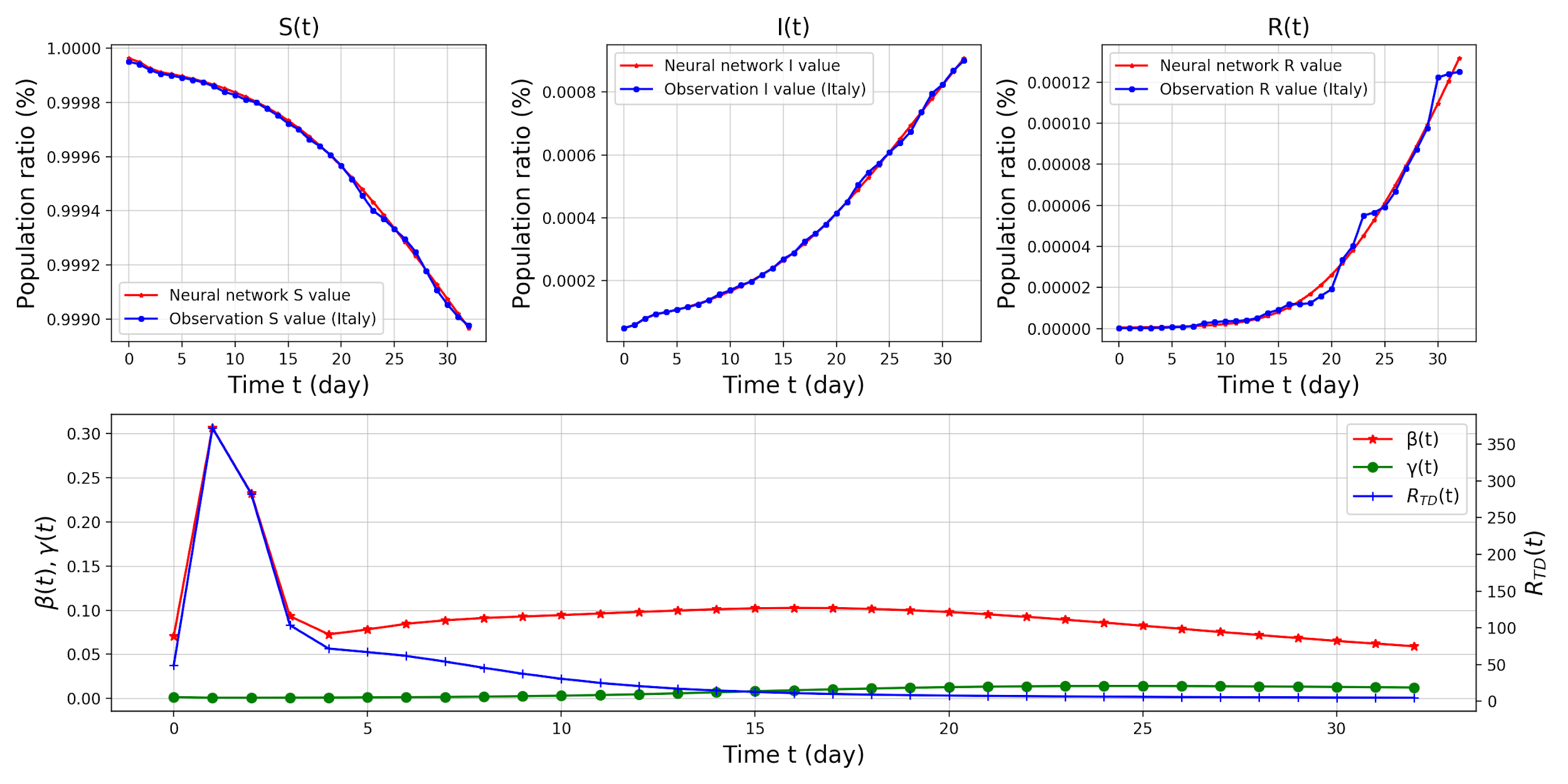


### Figure SM5. First row : SIR-model target values and relative errors. From 11, March 2020 (t = 0) to 12, April 2020 (t = 32.0) in Sweden. Red lines denote $S_{net}, I_{net}, R_{net}$ values for each graph, Green lines denote the observations, and Blue lines denote the RK4 results with the parameters $\beta_{net}$, and $\gamma_{net}$. Second row : SIR-model Parameter network values and R_TD_. From 11, March 2020 (t = 0) to 12, April 2020 (t = 32.0) in Sweden.


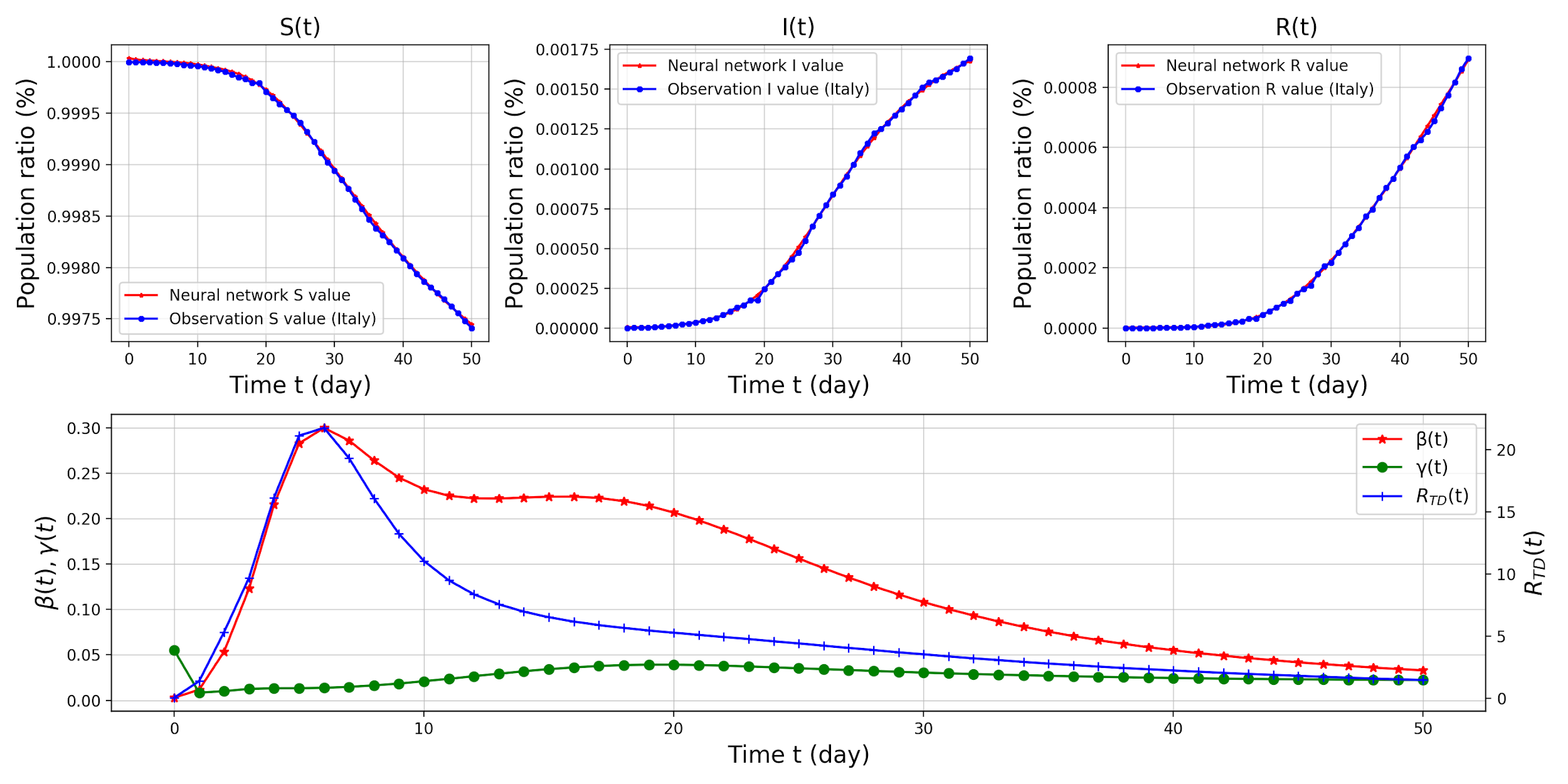


### Figure SM6. First row : SIR-model target values and relative errors. From 22, February 2020 (t = 0) to 12, April 2020 (t = 50.0) in Italy. Red lines denote $S_{net}, I_{net}, R_{net}$ values for each graph, Green lines denote the observations, and Blue lines denote the RK4 results with the parameters $\beta_{net}$, and $\gamma_{net}$. Second row : SIR-model Parameter network values and R_TD_. From 22, February 2020 (t = 0) to 12, April 2020 (t = 50.0) in Italy.


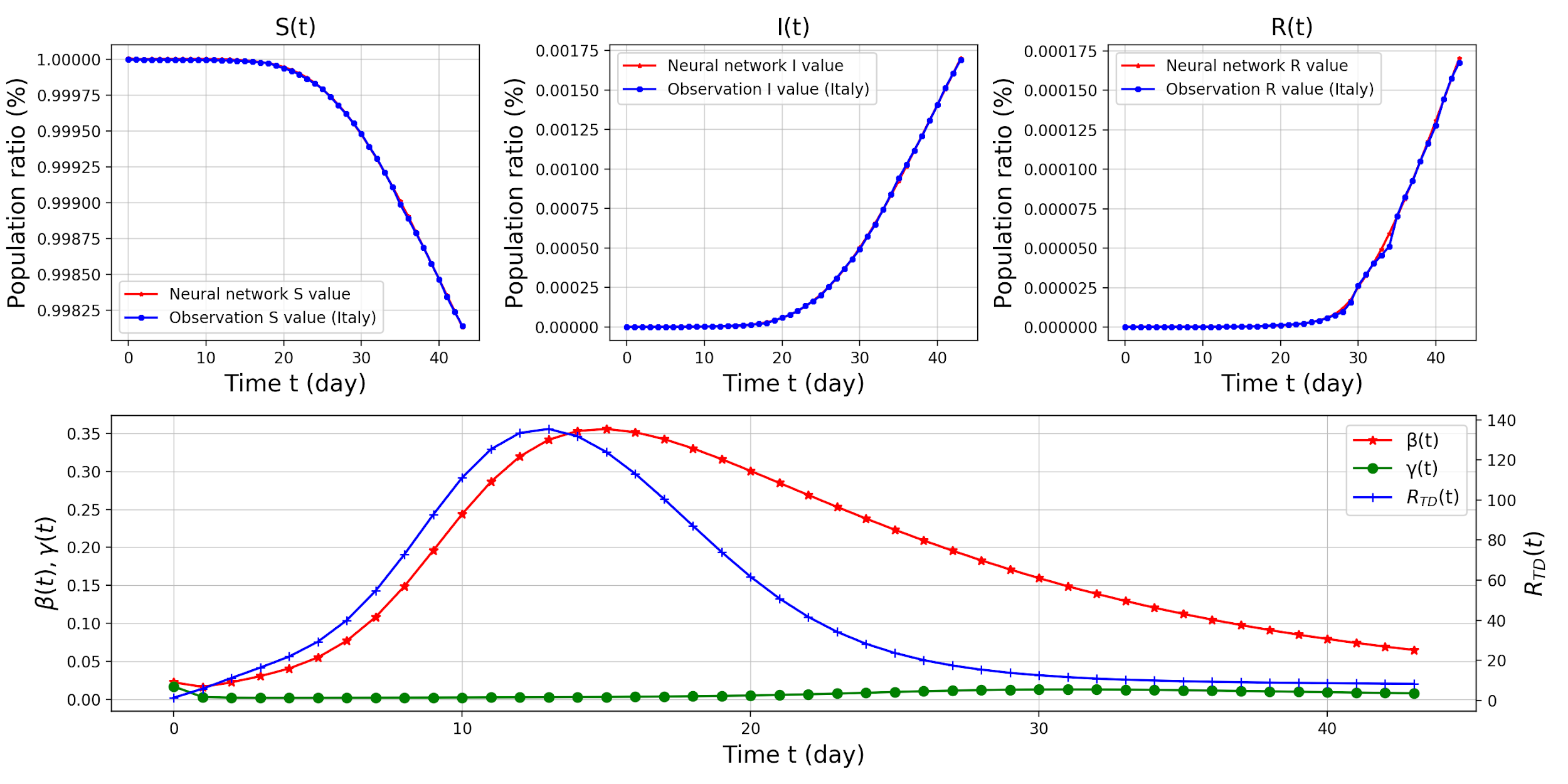


### Figure SM7. First row : SIR-model target values and relative errors. From 29, February 2020 (t = 0) to 12, April 2020 (t = 43.0) in the US. Red lines denote $S_{net}, I_{net}, R_{net}$ values for each graph, Green lines denote the observations, and Blue lines denote the RK4 results with the parameters $\beta_{net}$, and $\gamma_{net}$. Second row : SIR-model Parameter network values and R_TD_. From 29, February 2020 (t = 0) to 12, April 2020 (t = 43.0) in the US.

## Characteristic of $R_{TD}$


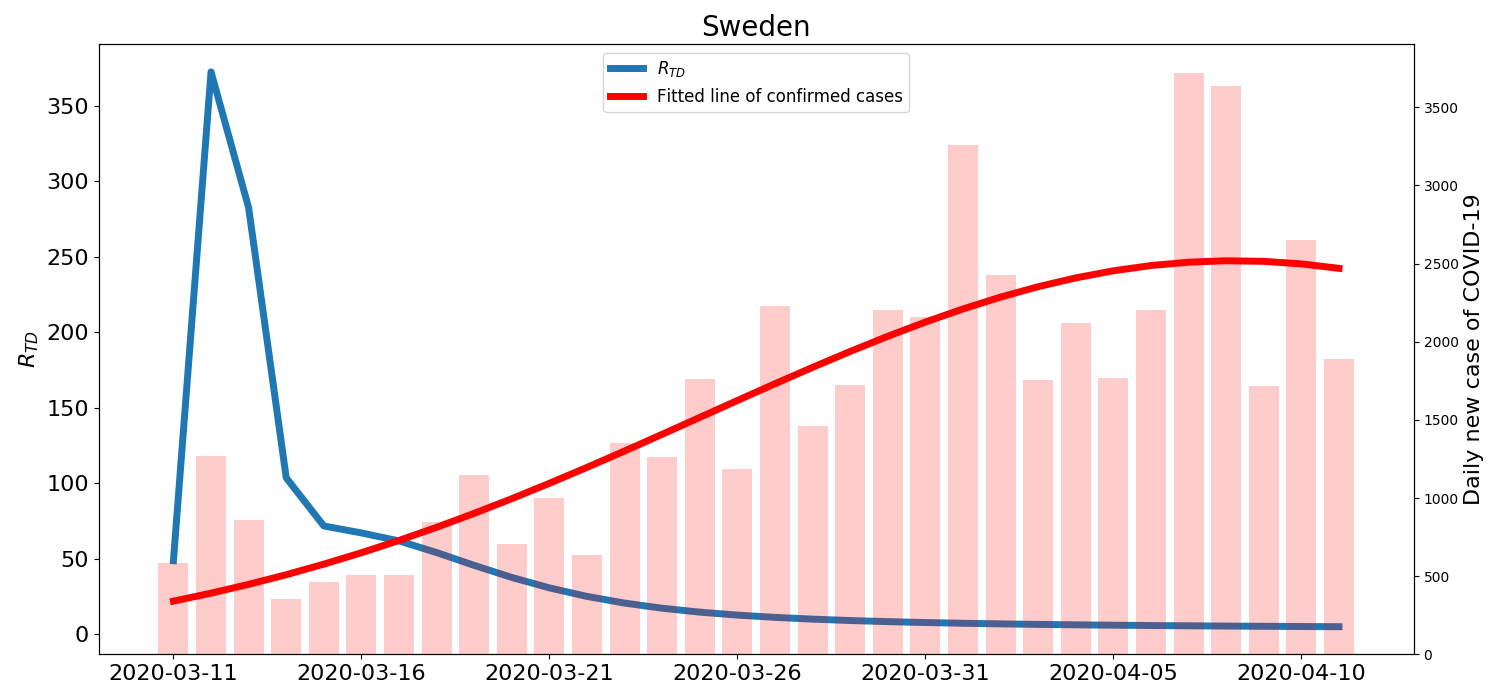


### Figure SM8. Comparison of R_TD_ and confirmed cases in Sweden.


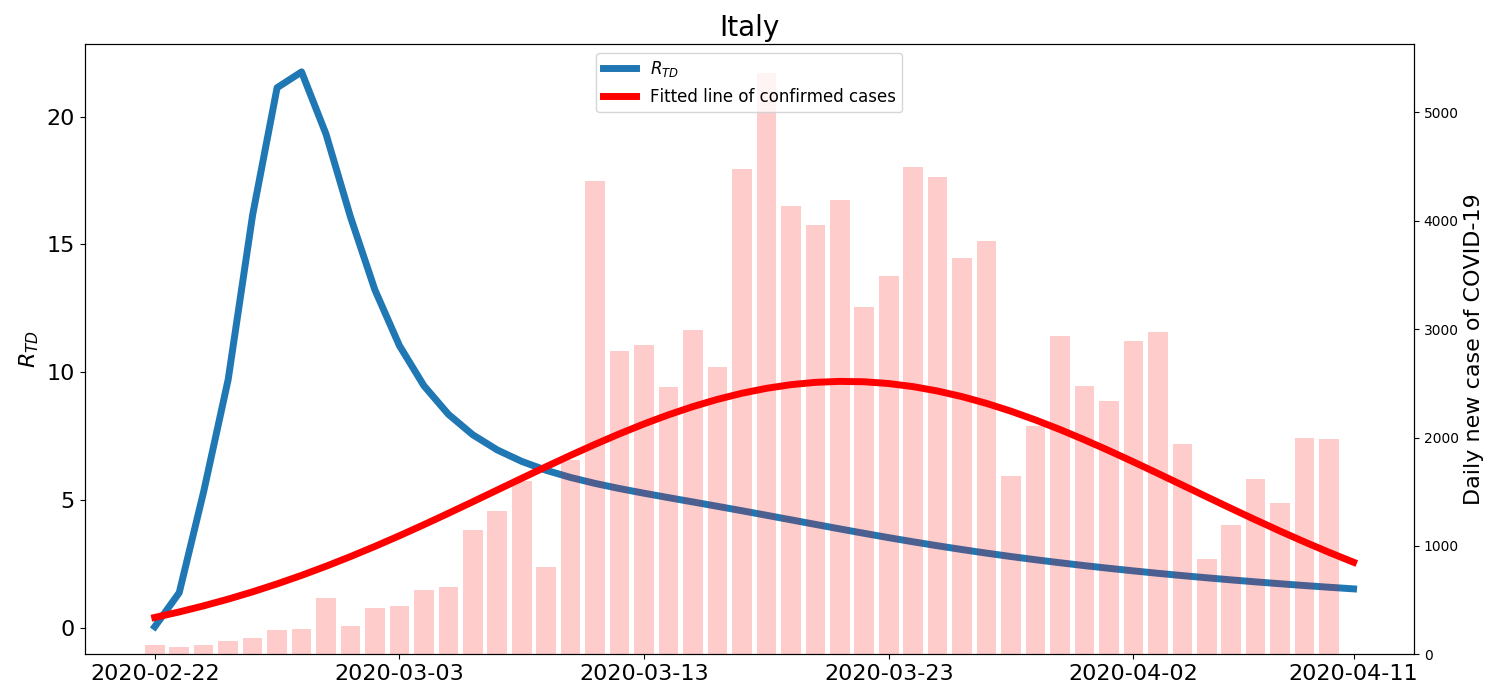


### Figure SM9. Comparison of R_TD_ and confirmed cases in Italy.

###
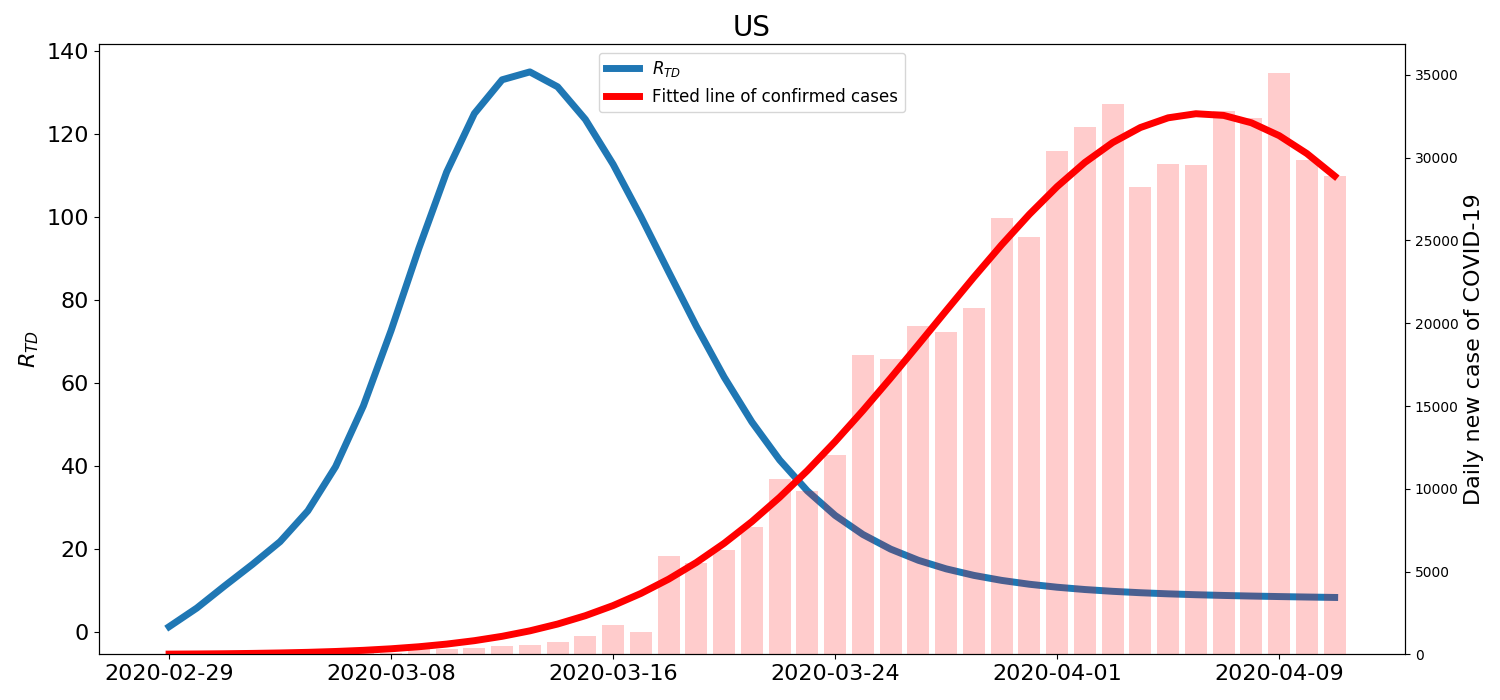


### Figure SM10. Comparison of R_TD_ and confirmed cases in US.

###

# References

1. [McCulloch WS, Pitts W. A logical calculus of the ideas immanent in nervous activity. Bull Math Biophys. 1943 Dec 1;5(4):115–33.](http://paperpile.com/b/7DpnHz/ZOAW)

2. [Cybenko G. Approximation by superpositions of a sigmoidal function. Math Control Signals Systems. 1989 Dec 1;2(4):303–14.](http://paperpile.com/b/7DpnHz/ib8o)

3. [Hornik K, Stinchcombe M, White H. Multilayer feedforward networks are universal approximators [Internet]. Vol. 2, Neural Networks. 1989. p. 359–66. Available from:](http://paperpile.com/b/7DpnHz/WURf) <http://dx.doi.org/10.1016/0893-6080(89)90020-8>

4. [Li X. Simultaneous approximations of multivariate functions and their derivatives by neural networks with one hidden layer. Neurocomputing. 1996 Aug 1;12(4):327–43.](http://paperpile.com/b/7DpnHz/odWC)

5. [Lagaris IE, Likas A, Fotiadis DI. Artificial neural networks for solving ordinary and partial differential equations. IEEE Trans Neural Netw. 1998;9(5):987–1000.](http://paperpile.com/b/7DpnHz/sBub)

6. [Lagaris IE, Likas AC, Papageorgiou DG. Neural-network methods for boundary value problems with irregular boundaries. IEEE Trans Neural Netw. 2000;11(5):1041–9.](http://paperpile.com/b/7DpnHz/nvlg)

7. [Berg J, Nyström K. A unified deep artificial neural network approach to partial differential equations in complex geometries. Neurocomputing. 2018 Nov 23;317:28–41.](http://paperpile.com/b/7DpnHz/8Eq2)

8. [Hwang HJ, Jang JW, Jo H, Lee JY. Trend to Equilibrium for the Kinetic Fokker-Planck Equation via the Neural Network Approach [Internet]. arXiv [math.NA]. 2019. Available from:](http://paperpile.com/b/7DpnHz/nWLh) <http://arxiv.org/abs/1911.09843>

9. [Raissi M, Perdikaris P, Karniadakis GE. Physics-informed neural networks: A deep learning framework for solving forward and inverse problems involving nonlinear partial differential equations. J Comput Phys. 2019 Feb 1;378:686–707.](http://paperpile.com/b/7DpnHz/T4OoR)

10. [Jo H, Son H, Hwang HJ, Kim E. Deep Neural Network Approach to Forward-Inverse Problems [Internet]. arXiv [math.NA]. 2019. Available from:](http://paperpile.com/b/7DpnHz/uafG) <http://arxiv.org/abs/1907.12925>
